# A quality improvement intervention to improve medium-term breastfeeding in moderate- and late-preterm infants

**DOI:** 10.1186/s13006-025-00751-3

**Published:** 2025-07-26

**Authors:** Katarina Berndt, Sabrina Holzapfel, Annika Dietz, Anna Badura, Ines Mack, Stefanie Bruhn, Sabine Stahl, Julia Preßler, Sven Wellmann

**Affiliations:** 1 Department of Neonatology, University Children’s Hospital Regensburg (KUNO), Hospital St. Hedwig of the Order of St. John, University of Regensburg, Regensburg, Germany; 2 Department of Paediatric Infectious Diseases and Vaccinology, University Children’s Hospital Basel (UKBB) and University of Basel, Basel, Switzerland

**Keywords:** Lactation, Breastfeeding, Preterm infant, Quality improvement, International board certified lactation consultants (IBCLCs)

## Abstract

**Background:**

Despite medical advancements, the rate of premature births remains at one in ten babies worldwide. Moderate and late preterm (MLPT, gestational age 32–36 weeks) infants constitute 80% of all preterm births and are at higher risk of short- and long-term complications compared to term infants. Breastfeeding helps to reduce these risks, but evidence on breastfeeding rates and success factors in MLPT infants is limited.

**Methods:**

A prospective intervention trial included a pre-intervention phase from June to September 2022 (comparison) and a post-intervention phase from June to October 2023 (intervention) at one tertiary academic hospital. Clinical parameters from pregnancy, delivery, and postnatal care were collected from MPLT infants and their mothers, including mid-term breastfeeding at infant´s four-month health check-up. Intervention was a quality improvement (QI) initiative involving staff training and parent education using an information platform (Neo-MILK app) with breastfeeding content. Primary outcome was breastfeeding rate at the age of four months after birth. Various secondary outcomes were defined, including growth sufficient exclusive breast milk feeding at 14 days after birth. Relative risks (RR) and 95% confidence intervals approximated from odds ratios obtained through univariate logistic regression to identify predictors of breastfeeding success.

**Results:**

Out of 170 eligible mothers of MLPT infants, 69 participated (36 intervention, 33 comparison group) with similar baseline characteristics. At four months of age (primary endpoint), 75% of the intervention group were breastfeeding compared to 48% of the comparison group (*p* = 0.023). Significant independent predictors of medium-term breastfeeding success were higher socioeconomic status (RR 1.16; 95% CI 1.01, 1.31), growth sufficient exclusive breast milk feeding and maternal self-efficacy, both measured at 14 days postpartum (RR 1.84; 95% CI 1.37, 2.01 and RR 1.04; 95% CI 1.02, 1.06, respectively). In contrast, delivery by cesarean section was associated with lower medium-term breastfeeding success (RR 0.21; 95% CI 0.07, 0.52).

**Conclusions:**

The implementation of a QI initiative, including breastfeeding education, early postpartum milk pumping and lactation support based on a common information platform for staff and parents was associated with increased medium-term breastfeeding success in MLPT infants. Despite early interventions, caesarean section remains a barrier to breastfeeding.

**Trial registration:**

The study is registered in the German Clinical Trials Register (DRKS00034762).

**Supplementary Information:**

The online version contains supplementary material available at 10.1186/s13006-025-00751-3.

## Background

Despite medical progress, the rate of premature births has not fallen in the last decade. One in ten babies worldwide is born prematurely [1]. Moderate and late preterm (MLPT, gestational age 32–36 weeks) infants constitute the majority of preterm births globally (80%) [2]. MLPT infants are born with lower birthweight compared to term born infants (on average 40% [3]) and are at higher risk of various neonatal complications including hypothermia, hypoglycaemia, respiratory distress, jaundice, and sepsis. This leads to frequent admissions to the neonatal intensive care unit (NICU) and separation of mother and child shortly after birth [4–6]. MLPT infants often have feeding difficulties that delay hospital discharge, increase readmission rate, and poorer rates of breastfeeding initiation and duration compared with term infants [7]. Moreover, MLPT infants have higher odds for long-term neurological and cardio metabolic disorders as compared to term born infants [8–11].

Breastfeeding is associated with reduced risk and severity of complications in preterm infants, particularly in very preterm and VLBW populations [12–15]. However, data on moderate- and late-preterm infants remain limited. Quality improvement (QI) interventions in neonatal intensive care units (NICUs) can support mothers and health care providers and have a positive impact on the success of breastfeeding in preterm infants. A recent systematic review and meta-analysis identified various QI interventions that significantly increased breastfeeding rates during hospitalisation [16]. The most common professional elements of a QI bundle included interdisciplinary work, education of hospital staff and parents, increased pump availability and early initiation of human milk expression, oropharyngeal colostrum administration, lactation consultant follow-up, skin-to-skin care and human milk management [16].

Previous studies primarily focused on preterm or very low birthweight infants during the hospital stay, leaving a gap in understanding medium and long-term breastfeeding rates and success factors in MLPT infants.

The aim of this study was to assess the status quo of breastfeeding rates in MLPT infants, to evaluate the impact of a QI bundle on medium-term breastfeeding success in clinical practice and to search for predictors and preventors of medium-term breastfeeding success in this population.

## Methods

### Study design

The study was conducted as a prospective intervention trial for quality improvement, featuring two recruitment phases. A pre-intervention phase lasting from June to September 2022 (comparison) and a post-intervention phase from June to October 2023 (intervention).

### Eligibility

Women eligible for the study were at least 18 years old, had given birth to a MLPT infant (32 + 0 to 36 + 6 weeks’ gestation) at the University perinatal center Regensburg, Hospital St. Hedwig of the Order of St. John, Germany, and were willing to participate up to three days after birth. Excluded were women with (1) multiple birth (due to differing lactation demands and feeding dynamics), (2) no wish to breastfeed after receiving counselling (informed decision), (3) German language skills less than level B1, (4) previous breast surgery, (5) glandular hypoplasia. Additional postnatal exclusion criteria were infants with (1) genetic syndromes, (2) osteoarticular disease or fractures (as these may limit positioning or early skin-to-skin contact), or (3) a priori palliative care.

### Intervention

The intervention consisted of the implementation and continuation of a QI bundle to support lactation and breastfeeding after completion of the pre-intervention phase with training for all clinical staff in the perinatal center. In detail, midwives, nurses and physicians performing outpatient service, delivery and post-delivery care in the Department of Obstetrics, Neonatology Intensive Care, the step-down Neonatology and the Well-baby nursery received training on breastfeeding support in MLPT infants, early milk expression, and communication strategies with parents.

To reach parents from the first visit in the perinatal center until discharge home, posters were displayed at central locations within the hospital and informational flyers were distributed. These included a QR code directing to the Neo-MILK App, a non-profit digital platform with multilingual and multimedia access for parents and professionals [17]. Parents were actively introduced to the app by staff during counselling sessions. In fact, the Neo-MILK platform served in the intervention period and afterwards as the single information platform for breastfeeding support.

In addition, a human donor milk bank was established in the hospital and went into operation two months after completion of the pre-intervention phase (November 2022).

### Outcome measures

All mothers enrolled in the study received a detailed pumping diary to be completed daily until day 14 after delivery, capturing e.g., daily pumping volumes and numbers of direct breastfeeding of their infants. Growth sufficient exclusively breast milk feeding was defined as sufficient weight gain with > 500 ml/day of pumped breast milk or exclusive breastfeeding or mixed breast milk feeding.

Further, mothers were asked to document breastfeeding status on a regular fashion beyond hospital discharge until four months after delivery and were approached by the study team by telephone one and four months after birth. Maternal knowledge was assessed through a single self-rated item. Maternal and neonatal demographic and clinical parameters, socio-economic status, mental health, and breastfeeding self-efficacy were assessed using the following validated questionnaires.

The Breastfeeding Self-Efficacy Scale–Short Form (BSES-SF) is a measure of breastfeeding self-efficacy and is considered suitable for clinical use to (a) identify high-risk breastfeeding mothers, (b) assess breastfeeding behavior and attitudes in order to individualize strategies, and (c) evaluate the effectiveness of different methods and guide program development [18]. Breastfeeding self-efficacy was assessed at 14 days and four months postpartum. Maternal mental health was assessed at 14 days and four months postpartum using the Edinburgh Postnatal Depression Scale (EPDS), which measures depressive symptoms over the past 7 days [19].

To assess the relationship between maternal breastfeeding practices, knowledge and attitudes towards breastfeeding and the mother’s socio-economic status (SES), the socio-economic index derived from the KiGGS-Study of the German Robert Koch-Institute was measured [20]. This index is based on three dimensions: education, occupation and income, with scores ranging from 1 to 7 calculated for each individual dimension and a total score ranging from 3 to 21. The SES index was included in the analysis as a metric (continous) variable in both univariable and multivariable regression models. Additionally, participants were categorized into SES groups based on the internal quintile distribution of the cohort: scores ≤ 15.1 were classified as low SES (1st quintile), scores between 15.1 and 18.7 as middle SES (2nd to 4th quintile), and scores > 18.7 as high SES (5th quintile). These categories reflect the relative SES distribution within our sample and are not directly comparable to national deprivation thresholds. The primary outcome of this study was the breastfeeding rate at the mandatory paediatric outpatient visit at four months of age, defined as any breastfeeding regardless of feeding method or formula supplementation. Secondary outcomes were growth sufficient exclusive breast milk feeding at 14 days after birth and factors influencing breastfeeding success.

Complete data were available for all 69 participants regarding the primary outcome (breastfeeding status at four months). For secondary outcomes, data availability was as follows: breastfeeding self-efficacy at 14 days postpartum (*n* = 56) and four months after discharge (*n* = 55); pumping diary at 14 days after birth (*n* = 67); and mental health status at both 14 days and four months postpartum (*n* = 57).

### Data management

QNOME, a secure web-based database developed in accordance with Good Clinical Practice (GCP) guidelines, was used for data and query management, monitoring and coding.

### Analysis

Data were analysed using IBM SPSS Statistics (version 29.0.0) with a significance level of *p* ≤ 0.05. Continuous variables are presented as median and Interquartile Range (IQR). Continuous variables are reported as mean ± standard deviation (SD) for normally distributed data and as median and interquartile range (IQR) for non-normally distributed data.Categorical data are presented as absolute (n) and percentage (%) frequencies. Continuous variables were first tested for normal distribution to determine the choice between parametric and non-parametric tests. Independent t-tests were used for normally distributed data, while non-normally distributed data were analyzed using the Mann-Whitney U test or the Wilcoxon test. The Chi-square test was used to analyze categorical variables. For small samples or low expected cell frequencies, Fisher’s exact test was used. For more complex categorical data, Monte Carlo simulation was used to calculate exact p-values. Odds ratios (OR) were estimated using univariate logistic regression analyses. Given the relatively high prevalence of the primary outcome, relative risks (RR) and corresponding 95% confidence intervals were approximated from ORs using the method proposed by 21 and are reported throughout this manuscript.

Multivariate logistic regression analysis was employed to identify independent predictors of breastfeeding success. Variables with a p-value < 0.1 in univariate analysis were initially considered. The final selection of covariates for multivariate logistic regression analysis was guided by goodness-of-fit parameters and included mode of delivery, socioeconomic status (SES), breastfeeding self-efficacy at 14 days postpartum, and growth sufficient exclusively breast milk feeding within the first 14 days. The trial was approved by the local ethics committee (Ethics Committee of the University of Regensburg) under the reference number 22-2923-101 on 25 May 2022 and registered in the German Clinical Trials Register on 23 July 2024 under the reference number DRKS00034762.

## Results

### Eligibility

**Fig. 1:**
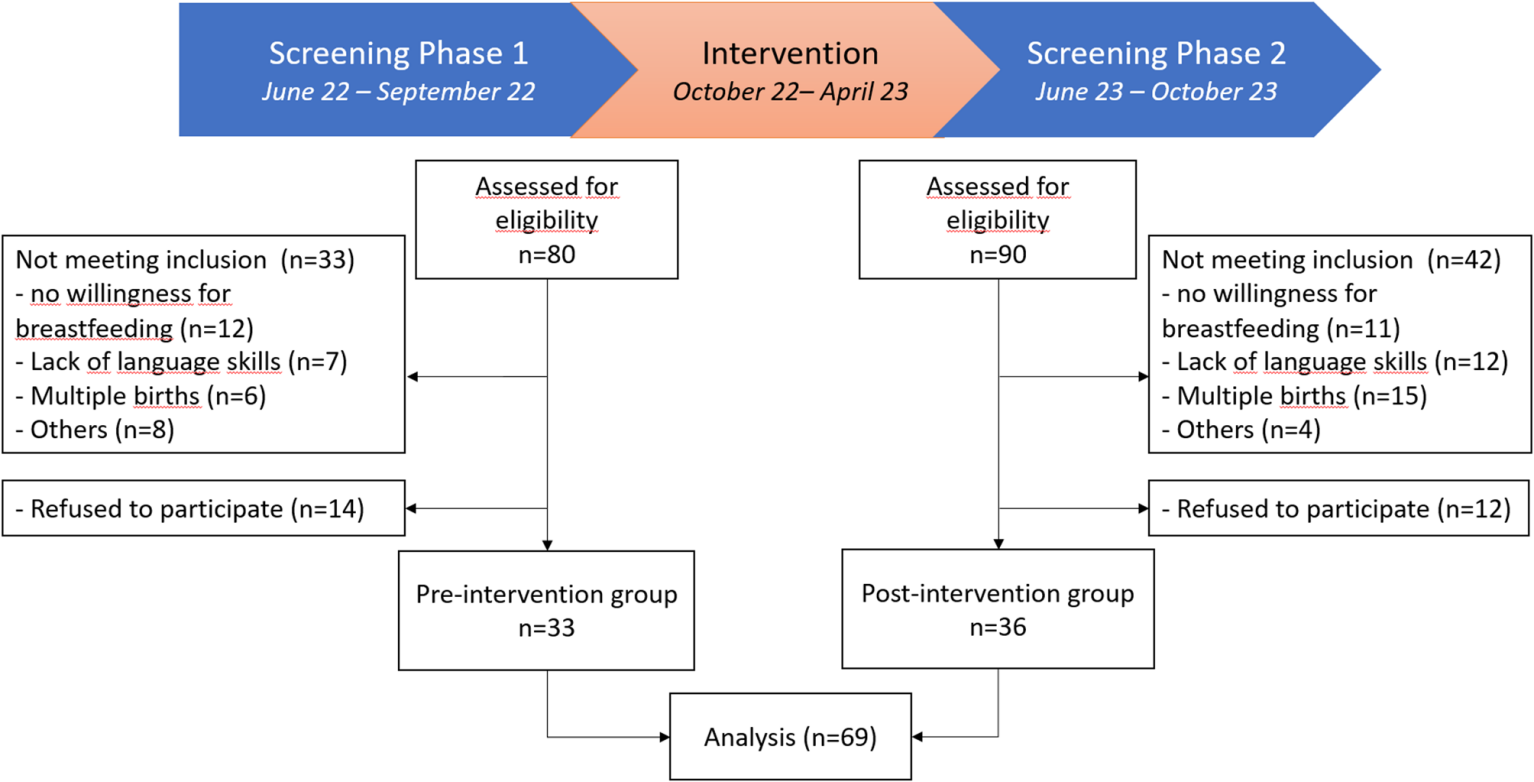
Study flow-chart

A total of 170 mothers were assessed for eligibility, resulting in 69 mothers who met the inclusion criteria and participated in the study. Of these, 33 were in the comparison group with assessment prior to the intervention and 36 were in the intervention group, assessed after QI implementation. The primary reasons for excluding mothers were unwillingness to breastfeed, multiple births, insufficient language skills and refusal to participate (Fig. 1).

### Baseline characteristics of the study cohort

The baseline characteristics of the cohort in our QI lactation study, which comprised a total of 69 mothers, were analysed to understand the demographic and clinical profiles of participants before and after the intervention. The baseline characteristics are presented in Table 1.

**Table 1:** Baseline characteristics and perinatal care variables

Characteristics		Total (*n* = 69)	Comparison (*n* = 33)	Intervention (*n* = 36)	*p*-value
Mother´s age, mean (SD) median (IQR)		31.5 (4.7) 32.0 (28.0;35.0)	30.9 (4.9) 31.0 (26.5;34.5)	32.0 (4.5) 33.0 (28.3;35.8)	0.344
First child, n (%)		44 (64)	19 (58)	25 (69)	0.742
Mother´s SES, mean (SD) median (IQR)		16.2 (2.5) 16.1 (15.3;18.7)	16.2 (2.7) 15.3 (15.1;18.7)	16.3 (2.4) 16.5 (15.3;18.5)	0.541
Skin to skin contact directly after birth, n (%)		51 (74)	23 (70)	28 (78)	0.445
Delivery mode vaginal, n (%)		37 (54)	14 (42)	23 (64)	0.074
Sex neonate female, n (%)		29 (42)	15 (46)	14 (39)	0.581
Gestational age (weeks), mean (SD) median (IQR)		35.3 (1.2) 35.7 (34.5;36.3)	35.2 (1.2) 35.7 (34.3;36.1)	35.4 (1.2) 35.9 (34.4;36.3)	0.431
Birth weight (g), mean (SD) median (IQR)		2516.7 (530.3) 2510 (2185;2846)	2447.6 (573.9) 2448 (2038;2809)	2579.9 (486.4) 2535 (2260;2858)	0.304
Hospital stay (days), mean (SD) median (IQR)		11.6 (10.4) 7.0 (4.0;17.0)	11.3 (9.5) 8.0 (4.0;17.0)	11.9 (11.2) 6.5 (4.0;16.5)	0.913
Maternal risk factors, n (%)	Obesity	10 (15)	4 (12)	6 (17)	0.737
	Diabetes	16 (23)	8 (24)	8 (22)	0.843
Neonatal risk factors, n (%)	Phototherapy	21 (30)	8 (24)	13 (36)	0.284
	Infusion, any	27 (39)	14 (42)	13 (36)	0.591

IQR: Interquartile Range. Comparison group is defined as the study population prior to QI implementation, intervention group is defined as the population after QI implementation. „Any infusion” includes intravenous fluids, parenteral nutrition, and medications (bolus or continuous). Continuous variables are shown as mean ± SD and median (IQR); p-values refer to the statistical test based on data distribution

Based on quintile distribution, 14.5% of participants were classified into the low SES group, 44.9% into the middle group, and 27.5% into the high SES group. There were no statistically significant differences in SES distribution between the intervention and comparison groups (*p* = 0.295).

### Association of QI measures with breastfeeding status and lactation performance within the first four months of life

At the time of discharge, almost all mothers (96%) of the overall cohort were breastfeeding their children, with 61% exclusively breastfeeding without giving any additional formula milk. Of the mothers who supplemented with formula, 46% mainly breastfed (about 2/3 breast milk and 1/3 formula), while 54% predominantly used formula (about 2/3 formula and 1/3 breast milk). The majority of the mothers who fed their children exclusively with breast milk gave them breast milk through breastfeeding (84%) and/or by bottle-feeding (67%), feeding tubes (7%) or alternative methods (2%). At day 14 postpartum, 39 mothers (57%) were exclusively feeding their infants with breast milk. Among them, 6 (15%) breastfed directly, 6 (15%) fed only pumped milk and 27 (69%) used a combination of both methods.

**Fig. 2:**
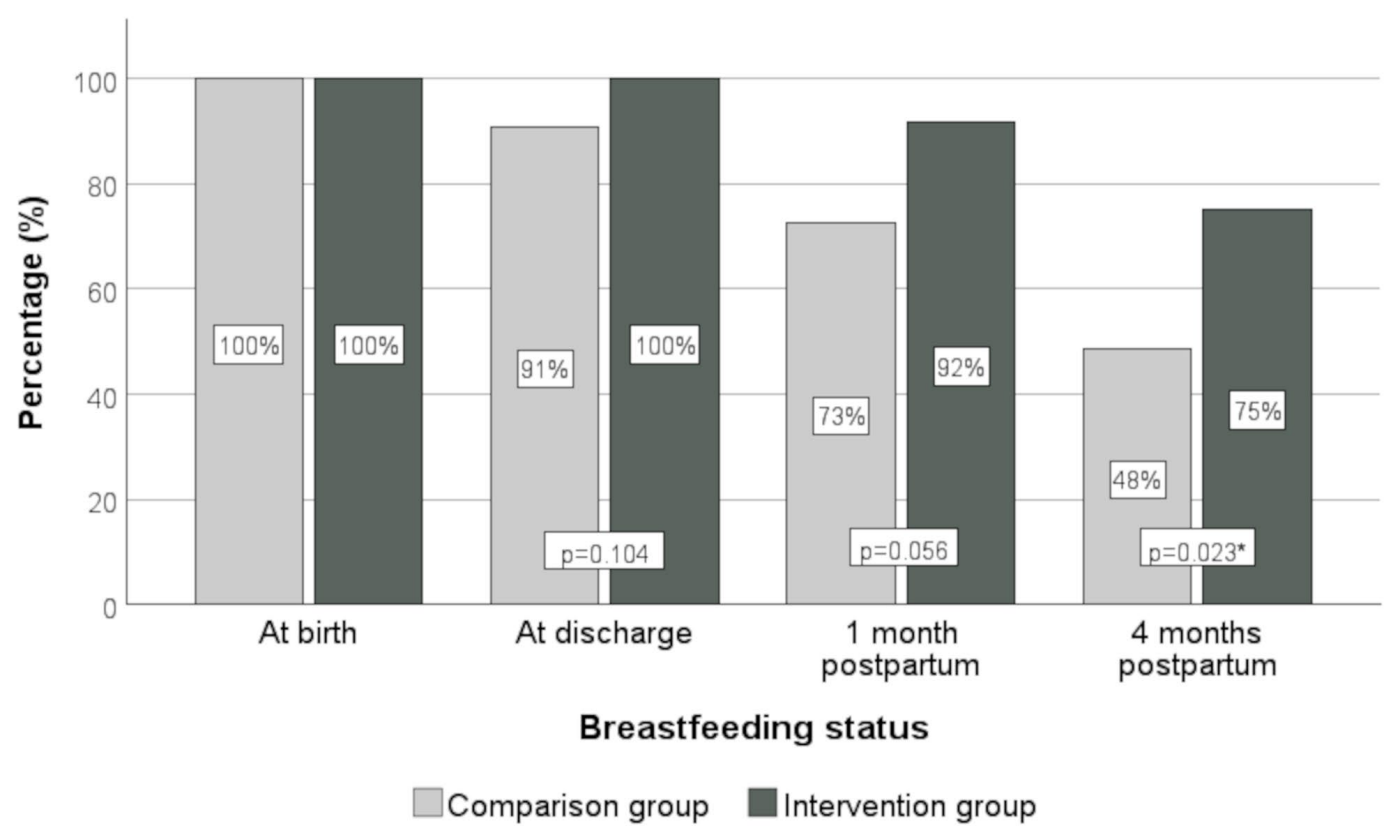
Progression of breastfeeding status within the first four months after birth

While 91% of the mothers in the comparison group were breastfeeding their infants at discharge, all mothers (100%) in the intervention group were breastfeeding at that time (*p* = 0.104). One month after discharge, 83% of mothers in the overall cohort were continuing to breastfeed, with higher rates in the intervention versus the comparisonl group (92% vs. 73%, *p* = 0.056). Four months after birth the breastfeeding status differed significantly, with 75% of mothers breastfeeding their babies in the intervention group compared to 48% in the comparison group (*p* = 0.023) (Fig. 2). At that timepoint, 59.7% of infants were exclusively breastfed. There was no significant difference between the comparison group (62%) and the intervention group (58%, *p* = 0.185). In addition, more mothers in the intervention group breastfed their babies directly at the breast, rather than using other methods like bottles or cups, compared to the comparison group (67% versus 42%, *p* = 0.043).

Breastfeeding self-efficacy was assessed at 2 weeks postpartum in 56 mothers with a median score of 73.5 (IQR 58.5; 81.25) for mothers in the intervention group compared to 68.5 (IQR 53.5; 79.75) when interviewed before the intervention (*p* = 0.582). These results did not change significantly when breastfeeding self-efficacy was assessed four months postpartum (*n* = 55 mothers), again with no group differences (*p* = 0.945).

### Maternal knowledge, perception of breastfeeding-support, and mental health in relation to QI measures

Mothers were asked to rate their knowledge on breastfeeding as “not at all”, “some” and “good”. Before intervention, 91% of the mothers reported “some” or “good knowledge” compared with 83.4% in the intervention group (*p* = 0.379).

Part of the intervention was to improve support for new mothers regarding breastfeeding techniques, including increased counselling by International Board Certified Lactation Consultants (IBCLCs). This implementation was confirmed successful as significantly more mothers in the intervention group reported receiving breastfeeding support (e.g. lactation counselling) compared to mothers interviewed before the QI campaign (63% compared to 30%; *p* = 0.010).

The maternal mental health status were available for 57 participants at both 14 days and four months postpartum. At both time points, there were no statistically significant differences in mental health status between mothers in the intervention group and those in the comparison group. At two weeks postpartum, the median score was 9.0 (IQR 4.0; 14.0) in the intervention group and 8.5 (IQR 6.0; 11.25) in the comparison group (*p* = 0.908). At four months postpartum, the median was 5.0 (IQR 4.0; 8.0) in the intervention group and 4.0 (IQR 1.0; 6.25) in the comparison group (*p* = 0.082).

### Factors associated with breastfeeding success

**Fig. 3:**
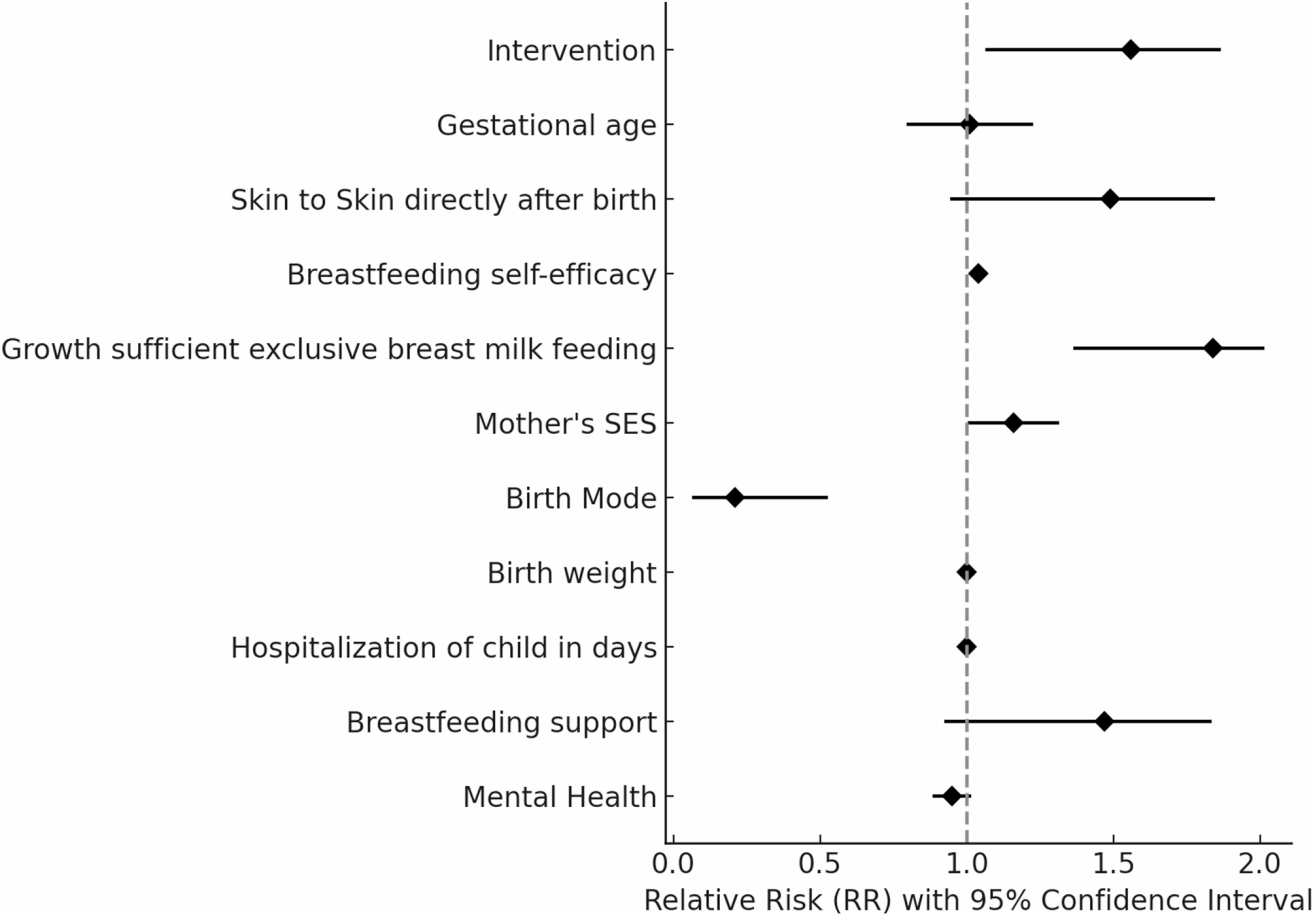
Predictors of breastfeeding four months postpartum. Forest plot showing estimated unadjusted relative risks (RR), based on odds ratios derived from univariate logistic regression analyses. Breastfeeding self-efficacy, growth sufficient exclusive breast milk feeding and mental health status were assessed at 14 days postpartum

Using univariate logistic regression analysis, several factors were identified as significant predictors of breastfeeding at four months postpartum (Fig. 3). These included mode of delivery, with caesarean section being associated with a lower likelihood of successful breastfeeding compared to vaginal births (RR 0.21; 95% CI 0.07, 0.52), and maternal socioeconomic status, with higher status mothers having a higher likelihood of breastfeeding at this time point (RR 1.16; 95% CI 1.01, 1.31). In addition, growth sufficient exclusive breast milk feeding at 14 days after birth (RR 1.84; 95% CI 1.37, 2.01) and higher levels of maternal self-efficacy (RR 1.04; 95% CI 1.02, 1.06) were significant factors influencing a positive breastfeeding outcome. Multivariate logistic regression analysis confirmed all these factors as independent significant factors. The intervention group was tested in an initial full model but did not remain statistically significant and was therefore excluded from the final model. The final selected multivariate regression model can be found in the Supplementary Material (Section S1).

## Discussion

The majority of preterm infants require medical support after birth, complicating breastfeeding initiation and medium to long-term success of breastfeeding. Here we demonstrate that a QI initiative based on a uniform information platform for health-care providers and parents was associated with increased medium-term breastfeeding rates until four months of age in MLPT infants. Moreover, we identified three predictors of medium-term breastfeeding success in MLPT infant: socioeconomic status, growth sufficient exclusive breast milk feeding and maternal self-efficacy at 14 days postpartum. Delivery by cesarean section was identified to prevent medium-term breastfeeding success in our study population. The intervention group, although relevant in unadjusted analyses, did not remain significant after adjustment and was excluded in the final model. This may indicate that the effect of the intervention was mediated by these individual factors, rather than acting as an independent predictor in the multivariable model. Breastfeeding is the first choice nutrition of infants. Key measures promoting breastfeeding during hospitalisation in term and preterm infants have already been identified [16, 22]. Although data on MLPT infants and medium- or long-term breastfeeding success remain limited, recent systematic reviews have begun to address this gap by identifying barriers, facilitators, and targeted interventions to support breastfeeding in this population [23, 24]. The European Society for Paediatric Gastroenterology Hepatology and Nutrition (ESPGHAN) has also highlighted that breastfeeding rates in MLPT infants are insufficient compared to term infants, underlining the need for targeted strategies in this group [7]. Our hospital provides guidelines on breastfeeding support and has a group of IBCLC trained staff for education and counselling, however effectiveness of these measures was not assessed yet. Therefore, we decided in the advent of opening a human donor milk bank and the expected need of donor milk, to execute a QI project on breastfeeding and taking advantage of the recently completed information platform Neo-MILK App by the University of Cologne.

The primary endpoint of our intervention study was breastfeeding rate at four months after birth, which was significantly higher in the intervention group (75% vs. 48%, *p* = 0.023). At the earlier study time points, there was a trend towards higher breastfeeding rates in the intervention group at the time of discharge home, which occurred on median at 8 days in the intervention group and at 6.5 days in the comparison group after birth. At the age of one month, there was a clear trend with a breastfeeding rates of 92% in the intervention group and 73% in the comparison group (*p* = 0.056). In summary, these results suggest that our QI interventions initiated early after birth and offering ongoing lactation and breastfeeding support by trained staff and the availability of the Neo-MILK App beyond discharge home, positively and sustainably influences breastfeeding practices. Thus, a real effect of the intervention seems to be more long-term and, in general, breastfeeding promotion programmes should not only aim at the immediate short-term effect, but also determine the medium to long-term outcome what is in line with previous investigations [25, 26].

More mothers in the intervention group achieved growth sufficient exclusive breast milk feeding at 14 days after birth (47% vs. 29%, respectively). Although this result was not statistically significant (*p* = 0.128), the higher rate of mothers with sufficient lactation suggests a trend towards better breastfeeding outcomes with improved support and reflects the findings of Hoban and colleagues [27]. Additionally, growth sufficient exclusive breast milk feeding within the first 14 days postpartum was an independent significant predictor of breastfeeding success four months after birth (RR 1.84; 95% CI 1.37, 2.01). Although no statistically significant difference in growth sufficient exclusive breast milk feeding was found between the groups, the intervention group did not remain significant in the multivariable model that included this variable. This suggests that growth sufficient exclusive breast feeding may have contributed to the observed association. As neither sufficient breast feeding nor skin-to-skin contact was significantly associated with the intervention, other components of the QI initiative—such as improved awareness among staff or more structured implementation of breastfeeding support measures—may have played a role. Delivery by caesarean section was associated with lower breastfeeding success (RR 0.21; 95% CI 0.07, 0.52), which emphasises the need for targeted support for both mothers and healthcare staff in this group, as well as the importance of providing information prior to birth. Although our study did not directly assess the timing of breastfeeding initiation, previous studies have shown that delayed initiation—often associated with caesarean delivery—can negatively affect breastfeeding outcomes [28, 29]. Although our data did not show a direct association between delayed initiation and breastfeeding success at four months [28], the results of our study showed that mode of delivery was associated with immediate and medium-term breastfeeding outcomes. Our finding of significantly lower breastfeeding success after caesarean section in MLPT infants despite broad breastfeeding support raises major concerns. Of course, we cannot exclude that our QI initiative did not meet the specific needs of women delivering MPLT infants by caesarean section. However, given the broad evidence that breastfeeding rates are lower after caesarean section irrespective of gestational age [30] there are most likely fetal and maternal processes inextricably linked to caesarean section ultimately complicating breastfeeding success per se [31, 32]. Therefore, health providers and health authorities should combine forces in limiting Caesarean section to medical needed indications and directing special support for breastfeeding to women after Caesarean section.

Higher socioeconomic status was associated with longer breastfeeding (RR 1.16; 95% CI 1.01, 1.31), potentially highlighting the role of resources and the complexity of social factors influencing breastfeeding success [33]. The importance of an individual needs analysis and personalized support should therefore be emphasised. Although we did not observe a significant difference in self-efficacy between groups in our study, previous research suggests that certain structured breastfeeding interventions—particularly those including individual counselling or skill-based support—can enhance maternal self-efficacy [34]. However, mothers’ early confidence in their ability to breastfeed was an important significant factor with positive effect on breastfeeding success four months postpartum (RR 1.04; 95% CI 1.02, 1.06).

Limitations of this study include a small sample size and the lack of control for confounding factors such as maternal health and cultural influences. The use of self-reported data captured by online questionnaires or in personal interviews could lead to bias. In addition, incomplete documentation in the daily pumping records cannot be ruled out with certainty. Further, the concurrent establishment of a human donor milk bank for extremely low birth weight infants (ELBW, birth weight < 1000 g) during the intervention phase may have contributed to improved breastfeeding outcomes and represents a potential confounding factor. However, as the milk bank accepted donations only from ELBW mothers with a surplus of milk, no overlap to the study population happened. However, the relative contribution of donor milk use versus QI measures remains unclear due to a lack of detailed data on milk supplementation patterns. Another limitation is the relatively low inclusion rate (< 50%), which may have introduced selection bias. Participants might have been more motivated, had stronger support systems, or differed socioeconomically from non-participants. This could affect the generalisability of our findings. Unfortunately, no detailed data were available on the characteristics of non-participants, which limits the ability to fully assess the direction and magnitude of potential selection bias. Furthermore, the one-year time gap between the comparison and intervention phases may have introduced temporal confounding, although major changes in clinical practice or patient population characteristics during this period are unlikely. Moreover, as this was a non-randomised study, no causal relationships can be inferred. In addition, as about three quarters of infants in our cohort were late preterm, the results may not be fully generalizable to moderate preterm infants, who may have different breastfeeding needs and challenges. Moreover, the limited sample size reduced the statistical power of the multivariable regression analysis, particularly for non-significant findings. Future research including multicentre RCTs should aim for larger and more diverse samples and objective measures of lactation to validate these findings and to specifically address the group of women giving birth by caesarean section.

## Conclusions

The results of this study indicate that targeted breastfeeding support by combining QI measures with an information platform for health-care providers and parents was associated with higher breastfeeding rates and duration in MLPT infants. Individual birth-related factors, the socio-economic context and the mothers’ confidence in their ability to breastfeed influence medium-term breastfeeding success.

## Electronic supplementary material

Below is the link to the electronic supplementary material.

Supplementary Material 1

Supplementary Material 2

Supplementary Material 3

Supplementary Material 4

## Data Availability

The datasets used and/or analysed during the current study are available from the corresponding author on reasonable request.

## Abbreviations

BSES-SF: Breastfeeding Self-Efficacy Scale–Short Form
CI: Confidence Interval
DRKS: Deutsches Register für Klinische Studien (German Clinical Trials Register)
EPDS: Edinburgh Postnatal Depression Scale
GCP: Good Clinical Practice
IBCLC: International Board Certified Lactation Consultants
IQR: Interquartile Range
MLPT INFANTS: Moderate and late preterm infants
NICU: Neonatal Intensive Care Unit
OR: Odds Ratio
QI: Quality Improvement
RR: Relative Risk
SES: Socio-Economic Status
