# Supplementary material for: A quality improvement intervention to improve medium-term breastfeeding in moderate- and late-preterm infants
Source: Int Breastfeed J. 2025 Jul 26;20:58. doi: 10.1186/s13006-025-00751-3 (PMC12296597; doi:10.1186/s13006-025-00751-3)
Supplement: Supplementary file 1 — Supplementary Material 1 [file 13006_2025_751_MOESM1_ESM.pdf]

Selbstsicherheit beim Stillen

Im folgenden bitten wir Sie Ihre persönliche Einschätzung zum Stillen abzugeben.

Frage 1

Ich kann sicherstellen, das mein Baby genug Milch bekommt.

Einfachauswahl

- Ich stimme überhaupt nicht zu
- Ich stimme eher nicht zu
- Ich stimme teilweise zu
- Ich stimme eher zu
- Ich stimme voll und ganz zu

Frage 2

Ich kann die Stillsituation (Abpumpen und tatsächliches Stillen) ähnlich erfolgreich bewältigen wie andere herausfordernde Aufgaben

Einfachauswahl

- Ich stimme überhaupt nicht zu
- Ich stimme eher nicht zu
- Ich stimme teilweise zu
- Ich stimme eher zu
- Ich stimme voll und ganz zu

Frage 3

Ich kann mein Kind stillen ohne Säuglingsmilch als Ergänzung zu verwenden.

Einfachauswahl

- Ich stimme überhaupt nicht zu
- Ich stimme eher nicht zu
- Ich stimme teilweise zu
- Ich stimme eher zu
- Ich stimme voll und ganz zu

Frage 4

Ich kann sicherstellen, dass mein Kind während der ganzen Fütterung an der Brust gehalten wird.

Einfachauswahl

- Ich stimme überhaupt nicht zu
- Ich stimme eher nicht zu
- Ich stimme teilweise zu
- Ich stimme eher zu
- Ich stimme voll und ganz zu

APPROVAL COPY
For demonstration use only!

Frage 5

Ich kann das Stillen zu meiner Zufriedenheit durchführen.

Einfachauswahl

- ○ Ich stimme überhaupt nicht zu
- ○ Ich stimme eher nicht zu
- ○ Ich stimme teilweise zu
- ○ Ich stimme eher zu
- ○ Ich stimme voll und ganz zu

Frage 6

Ich schaffe es zu stillen, auch wenn mein Kind weint.

Einfachauswahl

- ○ Ich stimme überhaupt nicht zu
- ○ Ich stimme eher nicht zu
- ○ Ich stimme teilweise zu
- ○ Ich stimme eher zu
- ○ Ich stimme voll und ganz zu

Frage 7

Ich möchte auch weiterhin stillen.

Einfachauswahl

- ○ Ich stimme überhaupt nicht zu
- ○ Ich stimme eher nicht zu
- ○ Ich stimme teilweise zu
- ○ Ich stimme eher zu
- ○ Ich stimme voll und ganz zu

Frage 8

Ich stille auch in der Gegenwart von Familienangehörigen entspannt

Einfachauswahl

- ○ Ich stimme überhaupt nicht zu
- ○ Ich stimme eher nicht zu
- ○ Ich stimme teilweise zu
- ○ Ich stimme eher zu
- ○ Ich stimme voll und ganz zu

Frage 9

Ich bin mit meinem Stillen zufrieden .

Einfachauswahl

- ○ Ich stimme überhaupt nicht zu
- ○ Ich stimme eher nicht zu
- ○ Ich stimme teilweise zu
- ○ Ich stimme eher zu
- ○ Ich stimme voll und ganz zu

APPROVAL COPY
For demonstration use only!

Frage 10

Ich komme damit klar, dass Stillen und Abpumpen zeitaufwändig sein kann.

Einfachauswahl

- ○ Ich stimme überhaupt nicht zu
- ○ Ich stimme eher nicht zu
- ○ Ich stimme teilweise zu
- ○ Ich stimme eher zu
- ○ Ich stimme voll und ganz zu

Frage 11

Ich beende erst das Stillen auf der einen Seite, bevor ich meinem Kind die andere Brust anbiete.

Einfachauswahl

- ○ Ich stimme überhaupt nicht zu
- ○ Ich stimme eher nicht zu
- ○ Ich stimme teilweise zu
- ○ Ich stimme eher zu
- ○ Ich stimme voll und ganz zu

Frage 12

Ich stille mein Kind zu jeder Mahlzeit.

Einfachauswahl

- ○ Ich stimme überhaupt nicht zu
- ○ Ich stimme eher nicht zu
- ○ Ich stimme teilweise zu
- ○ Ich stimme eher zu
- ○ Ich stimme voll und ganz zu

Frage 13

Ich schaffe mit den Stillbedürfnissen meines Kindes Schritt zu halten.

Einfachauswahl

- ○ Ich stimme überhaupt nicht zu
- ○ Ich stimme eher nicht zu
- ○ Ich stimme teilweise zu
- ○ Ich stimme eher zu
- ○ Ich stimme voll und ganz zu

Frage 14

Ich weiß beim Stillen wann mein Kind satt ist.

Einfachauswahl

- ○ Ich stimme überhaupt nicht zu
- ○ Ich stimme eher nicht zu
- ○ Ich stimme teilweise zu
- ○ Ich stimme eher zu
- ○ Ich stimme voll und ganz zu

APPROVAL COPY
For demonstration use only!

Frage 15

Ich schaffe es genügend Milch für mein Kind abpumpen.

Einfachauswahl

- Ich stimme überhaupt nicht zu
- Ich stimme eher nicht zu
- Ich stimme teilweise zu
- Ich stimme eher zu
- Ich stimme voll und ganz zu

Frage 16

Ich erhalte beim Stillen Hilfe wenn ich sie brauche.

Einfachauswahl

- Ich stimme überhaupt nicht zu
- Ich stimme eher nicht zu
- Ich stimme teilweise zu
- Ich stimme eher zu
- Ich stimme voll und ganz zu

Frage 17

Ich weiß wann mein Kind hungrig ist.

Einfachauswahl

- Ich stimme überhaupt nicht zu
- Ich stimme eher nicht zu
- Ich stimme teilweise zu
- Ich stimme eher zu
- Ich stimme voll und ganz zu

Frage 18

Ich kann vom Abpumpen zum Stillen übergehen oder mein Baby ganz stillen.

Einfachauswahl

- Ich stimme überhaupt nicht zu
- Ich stimme eher nicht zu
- Ich stimme teilweise zu
- Ich stimme eher zu
- Ich stimme voll und ganz zu

APPROVAL COPY
For demonstration use only!
