# Supplementary material for: A quality improvement intervention to improve medium-term breastfeeding in moderate- and late-preterm infants
Source: Int Breastfeed J. 2025 Jul 26;20:58. doi: 10.1186/s13006-025-00751-3 (PMC12296597; doi:10.1186/s13006-025-00751-3)
Supplement: Supplementary file 2 — Supplementary Material 2 [file 13006_2025_751_MOESM2_ESM.pdf]

Fragen zu Ihrer momentanen Lebenssituation

Nun zum Abschluss noch einige Fragen zu Ihnen

Frage 1

Welchen Schulabschluss haben Sie?

Einfachauswahl

- ○ Hauptschulabschluss/Volksschulabschluss
- ○ Realschulabschluss (Mittlere Reife)
- ○ Abschluss Polytechnische Oberschule (POS. 10. Klasse)
- ○ Fachhochschulreife (Abschluss einer Fachoberschule)
- ○ Abitur (Gymnasium bzw. EOS)
- ○ Anderer Schulabschluss
- ○ (Noch) keinen Schulabschluss

Frage 2

Haben Sie eine abgeschlossene Berufsausbildung? Wenn ja, welche?

Einfachauswahl

- ○ Lehre (beruflich-betriebliche Ausbildung)
- ○ Berufsschule, Handelsschule (beruflich-schulische Ausbildung)
- ○ Fachschule (z.B. Meister-Technikerschule, Berufs- oder Fachakademie)
- ○ Fachhochschule, Ingenieurschule
- ○ Universität, Hochschule
- ○ Anderer Ausbildungsabschluss
- ○ kein beruflicher Abschluss (und auch nicht in der Ausbildung)
- ○ In beruflicher Ausbildung (Auszubildender, Student)

Frage 3

Welcher der folgenden Angaben zur Berufstätigkeit trifft auf Sie zu?

ⓘ Zurzeit...

Einfachauswahl

- ○ nicht berufstätig
- ○ arbeitslos
- ○ vorübergehende Freistellung (z.B. Erziehungsurlaub)
- ○ Teilzeit oder stundenweise berufstätig
- ○ voll berufstätig
- ○ auszubildender (z.B. Lehrling)

APPROVAL COPY
For demonstration use only!

Frage 4

In welcher beruflichen Stellung sind Sie hauptsächlich derzeit beschäftigt?

ⓘ Wenn sie nicht mehr berufstätig sind, nennen Sie bitte die berufliche Stellung, die Sie zuletzt innehatten.

Einfachauswahl

- ○ Arbeiter
- ○ Selbständiger (einschließlich mithelfender Familienangehöriger)
- ○ Angestellter
- ○ Beamter
- ○ Sonstige

Frage 5

Arbeiter – In welcher beruflichen Stellung sind Sie hauptsächlich derzeit beschäftigt?

ⓘ Wenn sie nicht mehr berufstätig sind, nennen Sie bitte die berufliche Stellung, die Sie zuletzt innehatten.

Einfachauswahl

- ○ Ungelernter Arbeiter
- ○ Angelernter Arbeiter (Teilqualifizierung)
- ○ Gelernter Arbeiter und Facharbeiter
- ○ Vorarbeiter, Kolonnenführer, Meister, Polier, Brigadier

Frage 6

Selbständiger – In welcher beruflichen Stellung sind Sie hauptsächlich derzeit beschäftigt?

ⓘ Wenn sie nicht mehr berufstätig sind, nennen Sie bitte die berufliche Stellung, die Sie zuletzt innehatten.

Einfachauswahl

- ○ Selbständiger Landwirt/Genossenschaftsbauer
- ○ Selbständiger Akademiker, freier Beruf
- ○ Sonstiger Selbständiger mit bis zu 9 Mitarbeitern
- ○ Sonstiger Selbständiger mit 10 und mehr Mitarbeitern
- ○ Mithelfender Familienangehöriger

Frage 7

Angestellter – In welcher beruflichen Stellung sind Sie hauptsächlich derzeit beschäftigt?

ⓘ Wenn sie nicht mehr berufstätig sind, nennen Sie bitte die berufliche Stellung, die Sie zuletzt innehatten.

Einfachauswahl

- ○ Angestellter mit einfacher Tätigkeit (z.B. Verkäufer, Kontorist, Stenotypist)
- ○ Angestellter mit qualifizierter Tätigkeit (z.B. Sachbearbeiter, Buchhalter, technischer Zeichner)
- ○ Industrie- und Werkmeister im Angestelltenverhältnis
- ○ Angestellter mit hochqualifizierter Tätigkeit oder Leitungsfunktion (z.B. wissenschaftlicher Mitarbeiter, Prokurist, Abteilungsleiter)
- ○ Angestellter mit umfassenden Führungsaufgaben (z.B. Direktor, Geschäftsführer, Vorstand)

APPROVAL COPY
For demonstration use only!

Frage 8

Beamter – In welcher beruflichen Stellung sind Sie hauptsächlich derzeit beschäftigt?

ⓘ Wenn sie nicht mehr berufstätig sind, nennen Sie bitte die berufliche Stellung, die Sie zuletzt innehatten.

Einfachauswahl

- ○ Einfacher Dienst
- ○ Mittlerer Dienst
- ○ Gehobener Dienst
- ○ Höherer Dienst

Frage 9

Sonstige– In welcher beruflichen Stellung sind Sie hauptsächlich derzeit beschäftigt?

ⓘ Wenn sie nicht mehr berufstätig sind, nennen Sie bitte die berufliche Stellung, die Sie zuletzt innehatten.

Einfachauswahl

- ○ (z.B. Auszubildender, Schüler, Student, Wehrpflichtiger, Zivildienstleistender, Praktikant)
- ○ Hausfrau/Hausmann

Frage 10

Wie hoch ist das durchschnittliche monatliche Haushaltseinkommen, d.h. das Nettoeinkommen, das alle Haushaltsmitglieder zusammen nach Abzug von Steuern und Sozialabgaben haben? (Einschließlich Erziehungsgeld und Kindergeld)

Einfachauswahl

- ○ Unter 500 €
- ○ 500 bis unter 750 €
- ○ 750 bis unter 1.000 €
- ○ 1.000 bis unter 1.250 €
- ○ 1.250 bis unter 1.500 €
- ○ 1.500 bis unter 1.750 €
- ○ 1.750 bis unter 2.000 €
- ○ 2.000 bis unter 2.250 €
- ○ 2.250 bis unter 2.500 €
- ○ 2.500 bis unter 3.000 €
- ○ 3.000 bis unter 4.000 €
- ○ 4.000 bis unter 5.000 €
- ○ 5.000 € und mehr

APPROVAL COPY
For demonstration use only!
