# Supplementary material for: A quality improvement intervention to improve medium-term breastfeeding in moderate- and late-preterm infants
Source: Int Breastfeed J. 2025 Jul 26;20:58. doi: 10.1186/s13006-025-00751-3 (PMC12296597; doi:10.1186/s13006-025-00751-3)
Supplement: Supplementary file 3 — Supplementary Material 3 [file 13006_2025_751_MOESM3_ESM.pdf]

Fragen zu Ihrer momentanen Belastung

Gerne würden wir wissen, wie Sie sich in den letzten 7 Tagen gefühlt haben. Bitte markieren Sie die Antworten durch ankreuzen des Kreises vor jeder Antwortzeile, welche für Sie am ehesten zutrifft.

In den letzten 7 Tagen:

Frage 1

konnte ich lachen und das Leben von der sonnigen Seite sehen

Einfachauswahl

- ○ so wie ich es immer konnte
- ○ nicht ganz so wie sonst immer
- ○ deutlich weniger als früher
- ○ überhaupt nicht

Frage 2

konnte ich mich so richtig auf etwas freuen

Einfachauswahl

- ○ so wie immer
- ○ etwas weniger als sonst
- ○ deutlich weniger als früher
- ○ überhaupt nicht

Frage 3

fühlte ich mich unnötigerweise schuldig, wenn etwas schief lief

Einfachauswahl

- ○ ja, meistens
- ○ ja, manchmal
- ○ nein, nicht so oft
- ○ nein, niemals

Frage 4

war ich ängstlich und besorgt aus nichtigen Gründen

Einfachauswahl

- ○ nein, überhaupt nicht
- ○ selten
- ○ ja, manchmal
- ○ ja, häufig

Frage 5

erschrak ich leicht bzw. reagierte panisch aus unerfindlichen Gründen

Einfachauswahl

- ○ ja, oft
- ○ ja, manchmal
- ○ nein, nicht oft
- ○ nein, überhaupt nicht

APPROVAL COPY
For demonstration use only!

Frage 6

überforderten mich verschiedene Umstände

Einfachauswahl

- ○ ja, die meiste Zeit war ich nicht in der Lage, damit fertig zu werden
- ○ ja, manchmal konnte ich damit nicht fertig werden
- ○ nein, die meiste Zeit konnte ich gut damit fertig werden
- ○ nein, ich wurde so gut wie immer damit fertig

Frage 7

war ich so unglücklich, dass ich nicht schlafen konnte

Einfachauswahl

- ○ ja, die meiste Zeit
- ○ ja, manchmal
- ○ nein, nicht sehr oft
- ○ nein, überhaupt nicht

Frage 8

habe ich mich traurig und schlecht gefühlt

Einfachauswahl

- ○ ja, die meiste Zeit
- ○ ja, manchmal
- ○ selten
- ○ nein, überhaupt nicht

Frage 9

war ich so unglücklich, dass ich geweint habe

Einfachauswahl

- ○ ja, die ganze Zeit
- ○ ja, manchmal
- ○ nur gelegentlich
- ○ nein, niemals

Frage 10

überkam mich der Gedanke, mir selbst Schaden zuzufügen

Einfachauswahl

- ○ ja, ziemlich oft
- ○ manchmal
- ○ kaum
- ○ niemals

APPROVAL COPY
For demonstration use only!
