# Supplementary material for: A quality improvement intervention to improve medium-term breastfeeding in moderate- and late-preterm infants
Source: Int Breastfeed J. 2025 Jul 26;20:58. doi: 10.1186/s13006-025-00751-3 (PMC12296597; doi:10.1186/s13006-025-00751-3)
Supplement: Supplementary file 4 — Supplementary Material 4 [file 13006_2025_751_MOESM4_ESM.docx]

**Supplementary material**

**S1. Multivariate Regression analysis of early predictors for breastfeeding success until 4 months postpartum**

| ***Dependent variable: Breastfeeding status 4 months postpartum (yes/no)*** | | | | | | | | | |
| --- | --- | --- | --- | --- | --- | --- | --- | --- | --- |
|  | | B | SE | Wald | df | p-value | Exp(B) | 95% Confidence interval for EXP(B) | |
|  |  |  |  |  |  |  |  | Lower | Upper |
|  | Socioeconomic status | ,560 | ,272 | 4,231 | 1 | ,040 | 1,750 | 1,027 | 2,984 |
|  | Milk production* | 3,193 | 1,511 | 4,467 | 1 | ,035 | 24,370 | 1,261 | 470,927 |
|  | Birth Mode (C-section) | -3,393 | 1,331 | 6,493 | 1 | ,011 | ,034 | ,002 | ,457 |
|  | Breastfeeding self-efficacy | ,105 | ,043 | 5,914 | 1 | ,015 | 1,111 | 1,021 | 1,209 |
|  | intercept | -13,725 | 5,558 | 6,097 | 1 | ,014 | ,000 |  |  |

Coefficients represent adjusted odds ratios. Model fit: Nagelkerke R^2^: 0.710.
*Milk production > 500 ml within 14 days after birth.

**S2. Questionnaires (German Version)**

**S2.1 Mental Health**

**S2.2 Socioeconomic Status**

**S2.3 Breastfeeding Self-Efficacy**

**S3. Estimation of relative risks (RR)**

To provide a more accurate and interpretable measure of association, relative risks (RRs) and 95% confidence intervals were approximated from odds ratios (ORs) obtained via univariate logistic regression. The approximation was carried out using the method proposed by Zhang and Yu (1998), which is appropriate when the outcome of interest is relatively common (incidence >10%).

**The formula applied is as follows:**

​
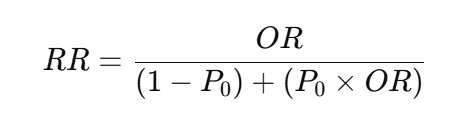


**Where:**

- RR = estimated relative risk
- OR = odds ratio from logistic regression
- P0​ = incidence of the outcome in the non-exposed (reference) group

**Reference:**

*Zhang J, Yu KF. What's the relative risk? A method of correcting the odds ratio in cohort studies of common outcomes. JAMA. 1998 Nov 18;280(19):1690–1691. doi:10.1001/jama.280.19.1690*

| **Predictor** | RR | 95% CI (lower) | 95% CI (upper) |
| --- | --- | --- | --- |
| Intervention | 1,56 | 1,07 | 1,86 |
| Gestational age | 1,01 | 0,8 | 1,22 |
| Skin-to Skin directly after birth | 1,49 | 0,95 | 1,84 |
| Breastfeeding self-efficacy | 1,04 | 1,02 | 1,06 |
| Expressed milk volume of 500 ml per day | 1,84 | 1,37 | 2,01 |
| Mother´s SES | 1,16 | 1,01 | 1,31 |
| Birth Mode | 0,21 | 0,07 | 0,52 |
| Birth weight | 1,00 | 0,99 | 1 |
| Hospitalization of child in days | 1,00 | 0,98 | 1,01 |
| Breastfeeding support | 1,47 | 0,93 | 1,83 |
| Mental Health | 0,95 | 0,89 | 1,01 |
